# Keratinocytes at the uppermost layer of epidermis might act as sensors of atmospheric pressure change

**DOI:** 10.1186/s13728-016-0052-2

**Published:** 2016-10-06

**Authors:** Mitsuhiro Denda

**Affiliations:** 1 Shiseido Global Innovation Center, 2-2-1, Hayabuchi, Tsuzuki-ku, Yokohama, 224-8558 Japan; 2 Japan Science Technology Agency CREST, Kawaguchi, Japan

**Keywords:** Climate influences, Pain, Peripheral nerve system, Peripheral circulation

## Abstract

It has long been suggested that climate, especially atmospheric pressure change, can cause health problems ranging from migraine to myocardial infarction. Here, I hypothesize that the sensory system of epidermal keratinocytes mediates the influence of atmospheric pressure change on the human physiological condition. We previously demonstrated that even subtle changes of atmospheric pressure (5–20 hPa) induce elevation of intracellular calcium level in cultured human keratinocytes (excitation of keratinocytes). It is also established that communication occurs between epidermal keratinocytes and peripheral nerve systems. Moreover, various neurotransmitters and hormones that influence multiple systems (nervous, cardiovascular, endocrine, and immune systems) are generated and released from epidermal keratinocytes in response to various external stimuli. Thus, I suggest that pathophysiological phenomena induced by atmospheric pressure changes might be triggered by epidermal keratinocytes.

## Background

Many reports indicate that atmospheric pressure change can have pathophysiological effects. For example, atmospheric pressure change might be associated with migraine and oral pain [1, 2], myocardial infarction and abdominal aortic aneurysm [3, 4], and alterations of the immune system [5, 6]. However, the mechanisms through which this might occur have not been clarified. Here, I hypothesize that epithelial cells in the uppermost layer of the epidermis, i.e., keratinocytes, first sense atmospheric pressure change and then induce systemic changes that could lead to pathophysiological events via production and release of multiple chemical mediators known to influence the nervous, cardiovascular, and immune system.

### Presentation of hypothesis

Epidermal keratinocytes sense atmospheric pressure change, and respond by producing and releasing chemical mediators that act systemically on multiple body systems.

### Testing the hypothesis

#### Responses of keratinocytes and other cells to atmospheric pressure change

**Fig. 1:**
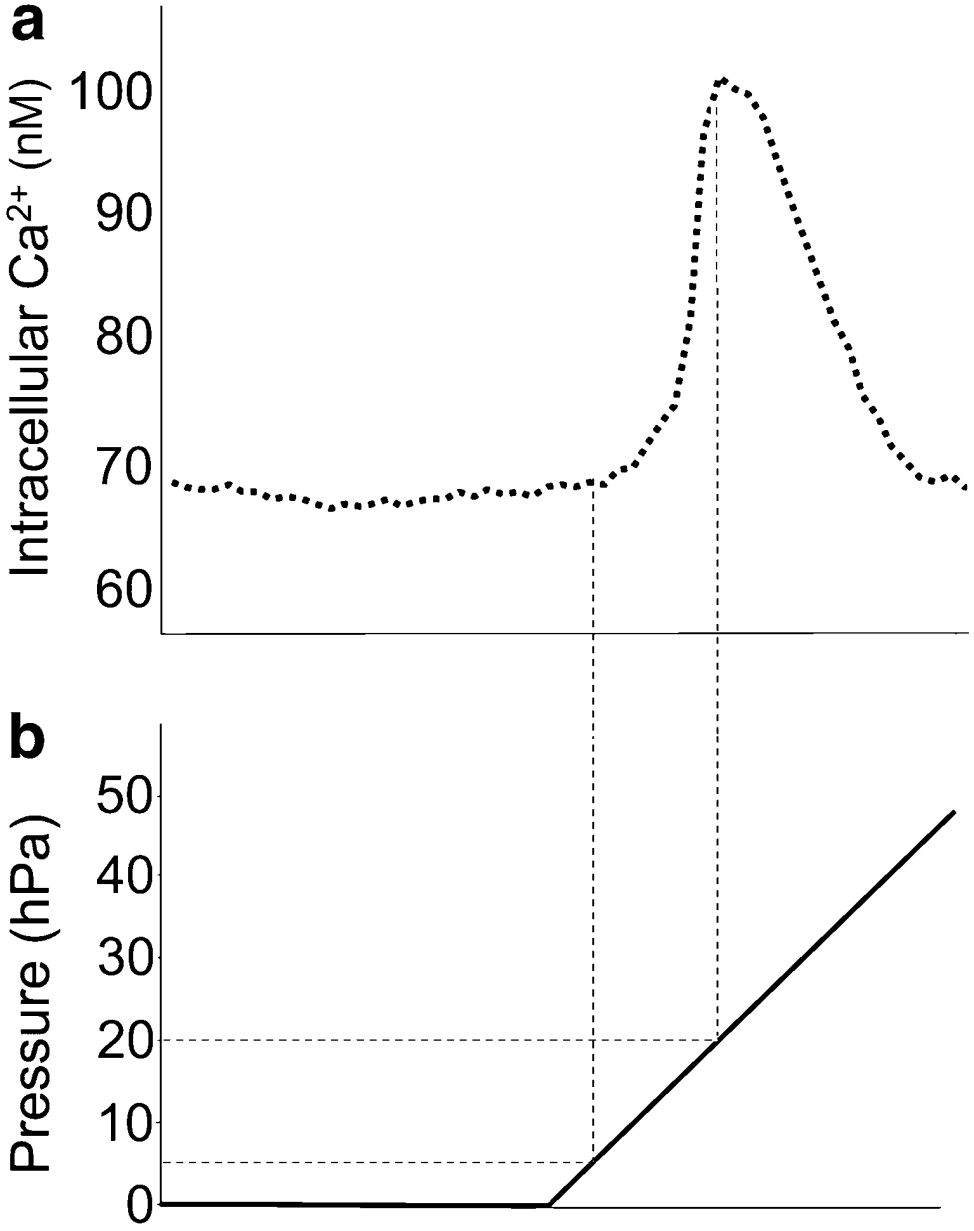
Representative profile of intracellular calcium level in differentiated keratinocytes (**a**) in relation to change of atmospheric pressure (**b**). For details, see Ref. [7]

We previously demonstrated that the intercellular calcium ion level ([Ca^2+^]_i_) of differentiated keratinocytes responds to changes of atmospheric pressure, and the threshold air-pressure increase from the initial level for inducing [Ca^2+^]_i_ response was 5–20 hPa (Fig. 1). In contrast, undifferentiated keratinocytes, fibroblasts, vein endothelial cells and neurons from dorsal root ganglion, all of which are components of human skin, showed no response in similar experiments. That is, only keratinocytes at the uppermost layer of the skin, i.e., at the interface between the body and the environment, function as sensors of atmospheric pressure change among all the cells in the skin, including peripheral nerve cells [7].

### Signals released from keratinocytes

We previously demonstrated that a variety of neurotransmitters and peptides are generated and released from keratinocytes after loading of external stress [8] (Table 1). Moreover, elevation of [Ca^2+^]_i_ in keratinocytes induced by mechanical stimuli induced elevation of [Ca^2+^]_i_ in peripheral nerve fibers. Although application of apyrase, an ATP-degrading enzyme, partially reduced the excitation of peripheral nerve fibers, other signal transducers could exist [9]. As described above, keratinocytes generate and release a variety of factors able to influence peripheral nerve systems. Thus, when keratinocytes are stimulated by atmospheric pressure change, signals might be passed to the peripheral and central nerve systems.

**Table 1:** Physiological factors produced by and released from epidermal keratinocytes

	References
Factors influencing the nervous system	
ATP	[14]
Prostaglandin E2	[16]
Glutamate	[8]
Dopamine	[8]
Factor influencing the cardiovascular system	
Nitric oxide (NO)	[17]
Factors influencing the endocrine and immune systems	
Proopiomelanocortin (POMC)	[18]
Corticotropin releasing hormone (CRH)	[18]
Cortisol	[19]
IL-1α	[20]
TNF-α	[20]
IL-6	[20]

### How pain might be induced by atmospheric pressure change?

Among receptors related to nociception, transient receptor potential cation channel subfamily V member 1 (TRPV1) is activated by heat (>43 °C), low pH (<6.6), and capsaicin [10], while P2X purinoceptor 3 (P2X3) is activated by ATP [11]; activation of both receptors induces the sensation of pain. These receptors were first discovered in the peripheral nerve system, but were later found to be functionally expressed in epidermal keratinocytes [12, 13]. A recent study suggested that activation of TRPV1 in keratinocytes induces nociception [14]. Moreover, ATP is released from keratinocytes in response to mechanical stress [9]. Prostaglandin, which could excite nerve systems, is also released from keratinocytes after external stimulation [15]. Thus, these pain receptors expressed in both epidermal keratinocytes and nerve systems might contribute to atmospheric pressure change-induced pain.

### How might the cardiovascular system be influenced by atmospheric pressure change?

We previously demonstrated that mechanical stress on the epidermis induces nitric oxide synthase (iNOS) in epidermal keratinocytes, leading to generation of nitric oxide (NO), which is released from the cells. We also observed that both peripheral blood vessels and lymphatic vessels were dilated by NO released from epidermal keratinocytes [16]. It had been long recognized that eNOS in endothelial cells is a significant source of NO, but we demonstrated that the level of NO synthesis in epidermal keratinocytes in skin was similar to that in endothelial cells [16]. Moreover, as mentioned above, differentiated keratinocytes could be stimulated by atmospheric pressure change, but cultured endothelial cells could not. These results suggested that atmospheric pressure change might be sensed by epidermal keratinocytes, causing them to synthesize and release NO, which, in turn, might influence the blood circulation.

### How might the immune and endocrine systems be influenced by atmospheric pressure change?

Previous studies indicate that all the components of the hypothalamo–pituitary–adrenal (HPA) axis are expressed in epidermal keratinocytes [17]. In addition, epidermal keratinocytes generate and release cortisol in response to environmental dryness [18]. Cytokines such as IL-1α, TNFα, and IL-6 are released from epidermal keratinocytes after external stimulation [19]. These hormones and cytokines are strongly associated with the immune system. So far, the effects of atmospheric pressure changes on generation of these factors in keratinocytes have not been clarified. Further studies will be needed to establish this, and to understand how climate changes influence our body condition.

### Implications of the hypothesis

Among the variety of cells in human skin, keratinocytes at the uppermost layer of the epidermis were the most sensitive to atmospheric pressure change, responding to a pressure change as small as 5–20 hPa. The barometric value was actual measured value, neither proportion nor percentages of atmospheric pressure. All experiments were carried out under normal atmospheric pressure (990–1024 kPa) in our laboratory (around 30 m above sea level). Our study was carried out with the closed vessel in the same room, and thus, percentage of each constituent gas was almost the same during the study. The resulting excitation of keratinocytes induces excitation of peripheral nerve systems. Moreover, factors released by excited keratinocytes are active on the cardiovascular, immune, and endocrine systems, and could potentially induce systemic pathophysiological changes.

Epidermal keratinocytes are also excited by high or low temperature [21] and environmental humidity [22]. Thus, keratinocytes could mediate systemic responses to a wide range of environmental changes. It has been long suggested that climate influences human physiology and mental condition, and I believe the present hypothesis provides a plausible mechanistic basis.

It is noteworthy that evolution of human skin has involved loss of body hair, and this might have enabled skin sensory systems to function more effectively. In the case of pressure sensing specifically, one could speculate that sensing pressure changes might have provided forewarning of weather changes, which could have been advantageous for hunting and survival.

In conclusion, we found that differentiated keratinocytes, which cover the uppermost surface of almost the whole body, were the most sensitive cells in the skin to atmospheric pressure change. Thus, I propose that keratinocytes at the uppermost layer of the epidermis serve as an interface between the body and the environment, and can cause systemic pathophysiological effects in response to environmental changes, such as atmospheric pressure change. This hypothesis should be readily amenable to testing in transgenic animals whose keratinocytes lack mechanical stimulation receptors.

## Abbreviations

TRP: transient receptor potential cation channel
P2X3: P2X purinoceptor 3
NO: nitric oxide
NOS: nitric oxide synthase
HPA: hypothalamo–pituitary–adrenal
